# Homotopic Commissural Projections of Area Prostriata in Rat and Mouse: Comparison With Presubiculum and Parasubiculum

**DOI:** 10.3389/fncir.2020.605332

**Published:** 2020-11-13

**Authors:** Chang-Hui Chen, Jin-Meng Hu, Sheng-Qiang Chen, Shi-Ming Liu, Song-Lin Ding

**Affiliations:** ^1^Key Laboratory of Neuroscience, School of Basic Medical Sciences, Institute of Neuroscience, The Second Affiliated Hospital, Guangzhou Medical University, Guangzhou, China; ^2^Allen Institute for Brain Science, Seattle, WA, United States

**Keywords:** prostriata, presubiculum, parasubiculum, interhemispheric connections, cre- dependent tracing, entorhinal cortex

## Abstract

Area prostriata in primates has recently been found to play important roles in rapid detection and processing of peripheral visual, especially fast-moving visual information. The prostriata in rodents was not discovered until recently and its connectivity is largely unknown. As a part of our efforts to reveal brain-wide connections of the prostriata in rat and mouse, this study focuses on its commissural projections in order to understand the mechanisms underlying interhemispheric integration of information, especially from peripheral visual field. Using anterograde, retrograde and Cre-dependent tracing techniques, we find a unique commissural connection pattern of the prostriata: its layers 2-3 in both hemispheres form strong homotopic commissural connections with few heterotopic projections to bilateral medial entorhinal cortex. This projection pattern is in sharp contrast to that of the presubiculum and parasubiculum, two neighbor regions of the prostriata. The latter two structures project very strongly to bilateral medial entorhinal cortex and to their contralateral counterparts. Our results also suggest the prostriata is a distinct anatomical structure from the presubiculum and parasubiculum and probably plays differential roles in interhemispheric integration and the balancing of spatial information between two hemispheres.

## Introduction

Area prostriata is a limbic cortex situated at the junction of the retrosplenial cortex (RS), dorsal presubiculum (PrSd; i.e., postsubiculum), parasubiculum (PaS) and mediodorsal part of the primary and secondary visual cortex (Sanides, 1969; Morecraft et al., 2000; Rockland, 2012; Ding, 2013). The prostriata was described 50 years ago (Sanides, 1969; Allman and Kaas, 1971) and has been confirmed in prosimian primates (Rosa et al., 1997), monkeys (Sousa et al., 1991; Barbas, 1993; Ding et al., 2003), and humans (Ding et al., 2016), but the rodent equivalent of this region was not identified until recently (Ding, 2013; Lu et al., 2020). This region in rodents was previously referred to as a part of the RS (i.e., area 29) (Blackstad, 1956; Haug, 1976; Slomianka and Geneser, 1991; Paxinos and Franklin, 2001; Paxinos and Watson, 2013; Preston-Ferrer et al., 2016), the triangular region of the PrSd (Honda et al., 2008) or a part of postsubiculum (Swanson, 2018) mainly based on connectivity data. We have identified this region as the prostriata based on anatomical location/topography, cytoarchitecture, molecular signature and connectivity (Ding, 2013; Lu et al., 2020) although useful functional data are not available in rodents.

The prostriata has been found to be important for the rapid analysis and integration of peripheral visual stimuli in monkeys (Rockland, 2012; Yu et al., 2012) and humans (Mikellidou et al., 2017; Tamietto and Leopold, 2018). This is consistent with our recent finding in rodents that the prostriata receives direct projections from primary visual cortex (V1), especially from its peripheral representation, i.e., medial V1 (Lu et al., 2020). Moreover, the prostriata in rodents appears to receive afferent projections from many other regions including subiculum (Sub), secondary (association) visual and auditory cortices (V2 and A2, respectively), presubiculum (PrS), anterior cingulate area (ACA), and anterior thalamic nuclei (ATN) (Ding, 2013; Ding et al., 2020; Lu et al., 2020; unpublished data). Projections from the ATN to prostriata was also reported in Tipaia (Conrad and Stumpf, 1975). All these afferent projections suggest that the prostriata is a critical region where different information from primary, secondary (association) cortices and limbic structures merge and integrate. The cortical outputs of the prostriata reach to V1 (Sousa et al., 1991), cingulate motor cortex (Morecraft et al., 2000), middle temporal visual area (Palmer and Rosa, 2006), orbitofrontal cortex and frontal pole (Barbas, 1993; Cavada et al., 2000; Burman et al., 2011) as well as to association auditory cortex (Falchier et al., 2010) in non-human primates. In rodents, some of the outputs of the prostriata reach to V1 and subcortical structures such as ventral lateral geniculate nucleus (VLG) and pretectal nuclei (PTN) (see Lu et al., 2020). However, it is unclear whether there are commissural connections between bilateral prostriata, and if so, how cells of origin and axon terminals of these connections are organized.

Commissural connections participate in processing and integration of information between two hemispheres (Restani and Caleo, 2016). It was reported that two of prostriata's adjoining areas, PrS and PaS, have strong commissural connections with contralateral medial entorhinal cortex (MEC) in rat (van Groen and Wyss, 1990; Honda et al., 2008; Preston-Ferrer et al., 2016). To our knowledge, commissural connections of the prostriata, PrS and PaS were not reported in mouse. In this study we aim to reveal the organization, origins and terminations of the commissural connections of the prostriata in rat and mouse in comparison with the PrS and PaS. Detailed information about the commissural connections of the prostriata, PrS and PaS are essential to our understanding of bilateral processing and integration of spatial information from peripheral environment.

## Materials and Methods

### Tracing Experiments in Rats

#### Animals

Twenty-five adult Sprague-Dawley (SD) rats of both sexes (280–310 g, Beijing Vital River Laboratory Animal Technology Co., Ltd.) living in an environment with controlled light and free access to food and water were used in this present study. All operations were performed after deep anesthesia to reduce pain. All experimental procedures were followed in accordance with the protocols that have been approved by the Institutional Animal Care and Use Committee.

#### Animal Surgery

Rats were anesthetized with sodium pentobarbital (40 mg/kg, i. p.). In order to expose the skull, the hair on the top of the rats was shaved and a 2 cm midline incision was made after disinfection. The rat's head was fixed on a stereotaxic frame, and the height of the nose clip was adjusted with the bregma and lambda in the horizontal plane. A small drill was used to make an appropriate hole in the rat's skull, and 0.1 μL 10% biotinylated dextran amine (BDA, 10,000 MW, ThemoFisher Scientific) or 4% Fluoro-Gold (FG, Fluorochrome) in saline was injected into the rat's brain through a 0.5 μL Hamilton syringe for 10 min. The coordinates of target brain region prostriata were determined based on the rat brain atlas (Paxinos and Watson, 2013). After injection, the syringe was left in place for 10 min and then slowly pulled out. Then the scalp was sutured and the rats were returned to their home cages after recovery on a hotbed.

#### Brain Preparation

7–10 days after the operation, the rats were anesthetized with sodium pentobarbital and then perfused transcardially with 0.9% saline and 4% paraformaldehyde (PFA) in chilled 0.1 M phosphate buffer (PB, pH 7.3) in sequence. The brains were removed from the skull, post-fixed in PFA at 4°C overnight and stored in 0.1 M PB (pH 7.3) containing 15 and 30% sucrose, in sequence, until each brain sank to the bottom of the container. The brains were then removed from solution and their two hemispheres were separated with a midline cut. Each hemisphere was cut into 40-μm-thick sagittal sections on a freezing microtome.

#### FG Tracing

Sections from the brains injected with FG were examined under an epifluorescent microscope (Leica DM6B) or stained with immunohistochemistry (IHC) according to standard procedures. For the IHC, the sections were rinsed in 0.1M PB three times, for 10 min each time, and then incubated in room temperature with 3% hydrogen peroxide for 10 min. After blocking in 5% BSA for 40 min the sections were incubated at 4°C overnight with solution containing 0.3% triton X-100 and primary antibody (rabbit anti-FG, AB153-I, 1:10000, Sigma-Aldrich). After that, the sections were incubated with the secondary antibody solution (biotinylated goat anti-mouse/rabbit IgG, Boster Biological Technology) and then the Streptavidin-Biotin Complex solution (SABC kit, Boster Biological Technology) for 60 min each. The sections were visualized by incubating the sections in PB containing 0.05% 3, 3-diaminobenzidine (DAB) and 0.01% hydrogen peroxide. Finally, the sections were rinsed and mounted on chrome alum and gelatin-coated slides, dehydrated in a graded series of ethanols, and coverslipped.

#### BDA Tracing

Sections from the brains injected with BDA were stained according to previously published method (Lu et al., 2020). Briefly, after washing with 0.1M PBS (PH 7.3), sections were incubated with 0.3% Triton X-100 in 0.1M PB (PH 7.3) for 60 min. Then the sections were rinsed again with 0.1M PB and incubated with Streptavidin-Biotin Complex solution (SABC kit, Boster Biological Technology) for 120 min. Finally, the sections were visualized using DAB, mounted, and coverslipped according the procedure mentioned previously.

#### Image Acquisition and Analysis

Images of the stained sections were captured using a scanner (Aperio CS2, Leica). Selected images were further processed in Adobe Photoshop CS5, including image cropping, brightness adjustment and picture placement and anatomical annotation.

### Tracing Experiments in Mice

Raw data on mouse tracing experiments ware derived from Allen Mouse Brain Connectivity (http://connectivity.brain-map.org/), and the specific protocols are available online (http://help.brainmap.org/display/mouseconnectivity/documentation). Briefly, a pan-neuronal rAAV vector expressing EGFP under control of a human synapsin I promoter (AAV2/1.pSynI.EGFP.WPRE.bGH) or Cre-dependent rAVV (AAV AAV2/1.pCAG.FLEX.EGFP.WPRE.bGH) were injected to target brain regions. Mice were anesthetized with 5% isoflurane and placed into a stereotaxic frame (Model# 1900, Kopf, Tujunga, CA). Each mouse received an AAV injection in the target regions using iontophoresis, which means currents were applied for iontophoresis of rAAV particles (3 μA, 7 s on/7 s off cycle, for 5 min), in accordance with the chosen coordinates for each target based on the mouse brain atlas (Paxinos and Franklin, 2001). After surgery, the mice recovered and survived for 21 days prior to sacrifice. The mice were perfused transcardially with 10 ml of saline (0.9% NaCl) followed by 50 ml of freshly prepared 4% PFA. The brains were stored in PBS with 0.1% sodium azide. For imaging, brains were placed in 4.5% oxidized agarose, transferred to a phosphate buffer solution, and placed in a grid-lined embedding mold for standardized orientation in an aligned coordinate space. Multiphoton image acquisition was accomplished by using the TissueCyte 1,000 system (TissueVision, Cambridge, MA). Selected images were downloaded and further processed in Adobe Photoshop 2020, as mentioned above.

## Results

### Borders and Layers of Prostriata, Presubiculum, and Parasubiculum

**Figure 1:**
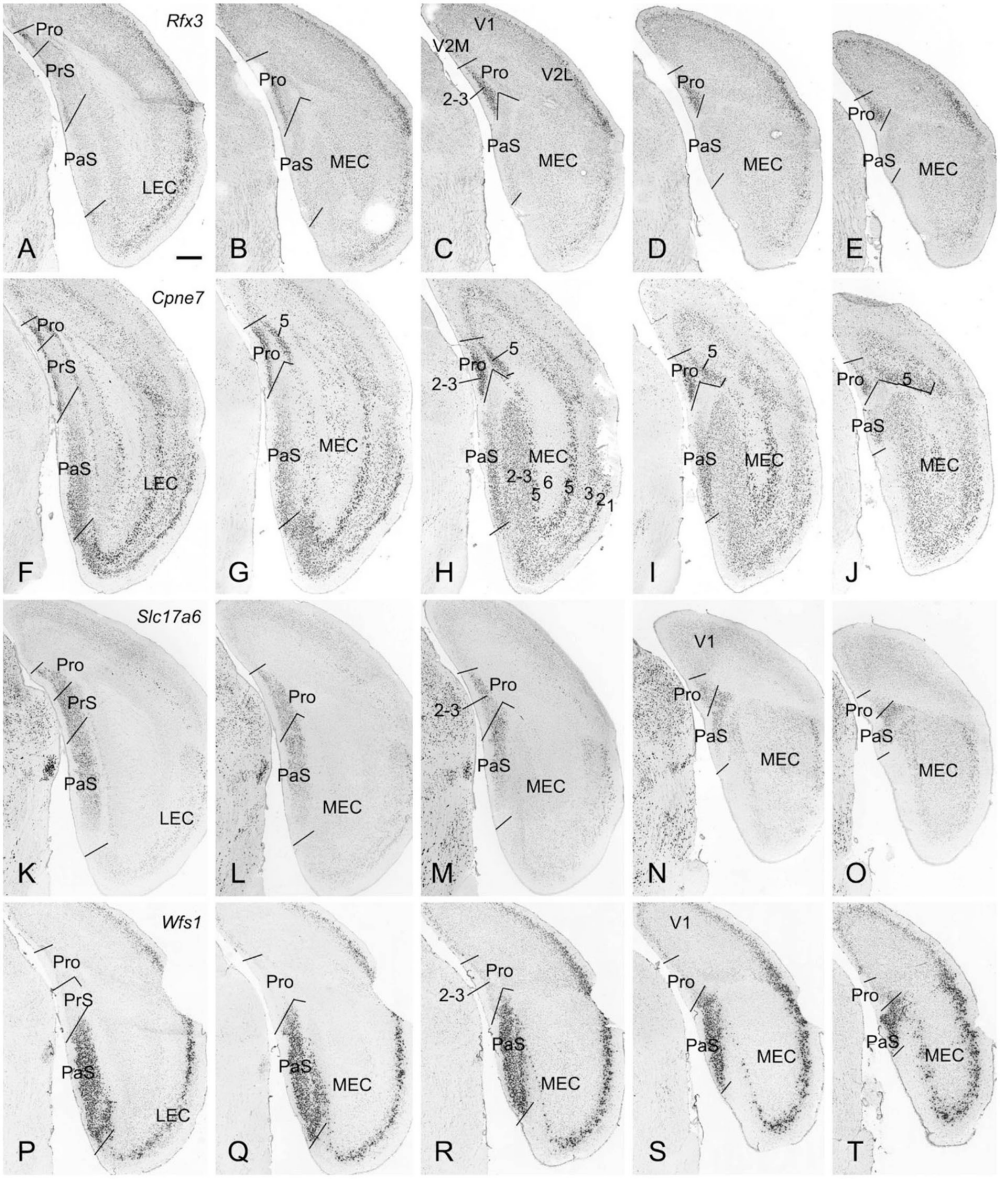
Borders and layers of the prostriata in mouse. Each row shows sequential rostral (left) to caudal (right) coronal sections. **(A–E)** *Rfx3*-ISH-stained sections showing the location, extent, and topography of layers 2-3 of the prostriata which have strong *Rfx3* expression. Weak and no *Rfx3* expression was seen in the PrS (layer 2) and PaS, respectively. **(F–J)** *Cpne7*-ISH-stained sections showing the location, extent and topography of the prostriata. Strong *Cpne7* expression was seen in layers 2–3 and 5 of the prostriata. Note the gradient of *Cpne7* expression in the PaS with strong and weak expression in ventral and dorsal PaS, respectively. Strong *Cpne7* expression was also observed in layers 2, 3, and 5 of the EC with few in layer 6. **(K–O)** *Slc17a6*-ISH-stained sections showing the strong expression in layers 2-3 of the prostriata, PrS and PaS with little expression in adjoining cortical regions. Note the gradient of *Slc17a6* expression in the PaS with strong and weak expression in dorsal and ventral PaS, respectively. **(P–T)** *Wfs1*-ISH-stained sections showing the location, extent, and topography of the PaS, which display strong *Wfs1* expression. Note the lack of expression in the prostriata and PrS. PrSd (i.e., postsubiculum) is seen in more rostral sections (not shown, but see Lu et al., 2020). Raw ISH data in this figure were downloaded from Allen Mouse Brain Atlas (mouse.brain-map.org). Bar: 420 μm in **A** (for all panels).

The borders and layers of the prostriata and adjoining RS, PrS, and PaS in both mouse and rat have been recently identified (Lu et al., 2020). Here, we briefly outline the borders and layers on some *in situ* hybridization (ISH)-stained sequential sections from mouse (www.brain-map.org) to provide some context for the locations of the prostriata and adjoining regions and some gene/Cre expression data related to the Cre-dependent tracing. For example, strong *Rfx3* expression in layers 2-3 of the prostriata clearly marks the extent of the prostriata since much weaker *Rfx3* expression was found in layer 2 of the PrS, RS and visual cortices (V1 and V2M) with no expression in the PaS (Figures 1A–E). Additionally, *Cpne7* expression in prostriata concentrates in layers 2-3 and 5. The pattern of *Cpne7* expression in layers 2-3 of the prostriata is similar to that of *Rfx3* expression. In layer 5, *Cpne7* expression in prostriata is very strong while that in adjoining cortices is much weaker, making layer 5 of the prostriata standing out (Figures 1F–J). Both *Rfx3* and *Cpne7* are expressed in layer 2 but not in layer 3 of the PrS. It is also worth mentioning that two subdivisions of the PaS [a and b; see Ding (2013)] can be distinguished based on expression differences between *Cpne7* (Figures 1F–J) and *Slc17a6* (Figures 1K–O). *Slc17a6* is a gene marker for superficial layers 2-3 of prostriata, PrS, and PaS (Figures 1K-O), with no expression in the deep layers. One exception is in the dorsal portion of PrSd where layer 2 shows few expression (not shown). *Wfs1* is an excellent gene marker for layers 2-3 of the PaS with no expression in the PrS and prostriata (Figures 1P–T). Finally, *Drd3* expression is distributed in layer 3 of the PrS and layers 2-3 of the PaS (not shown).

### Contralateral Projections of the Prostriata in Rats

**Figure 2:**
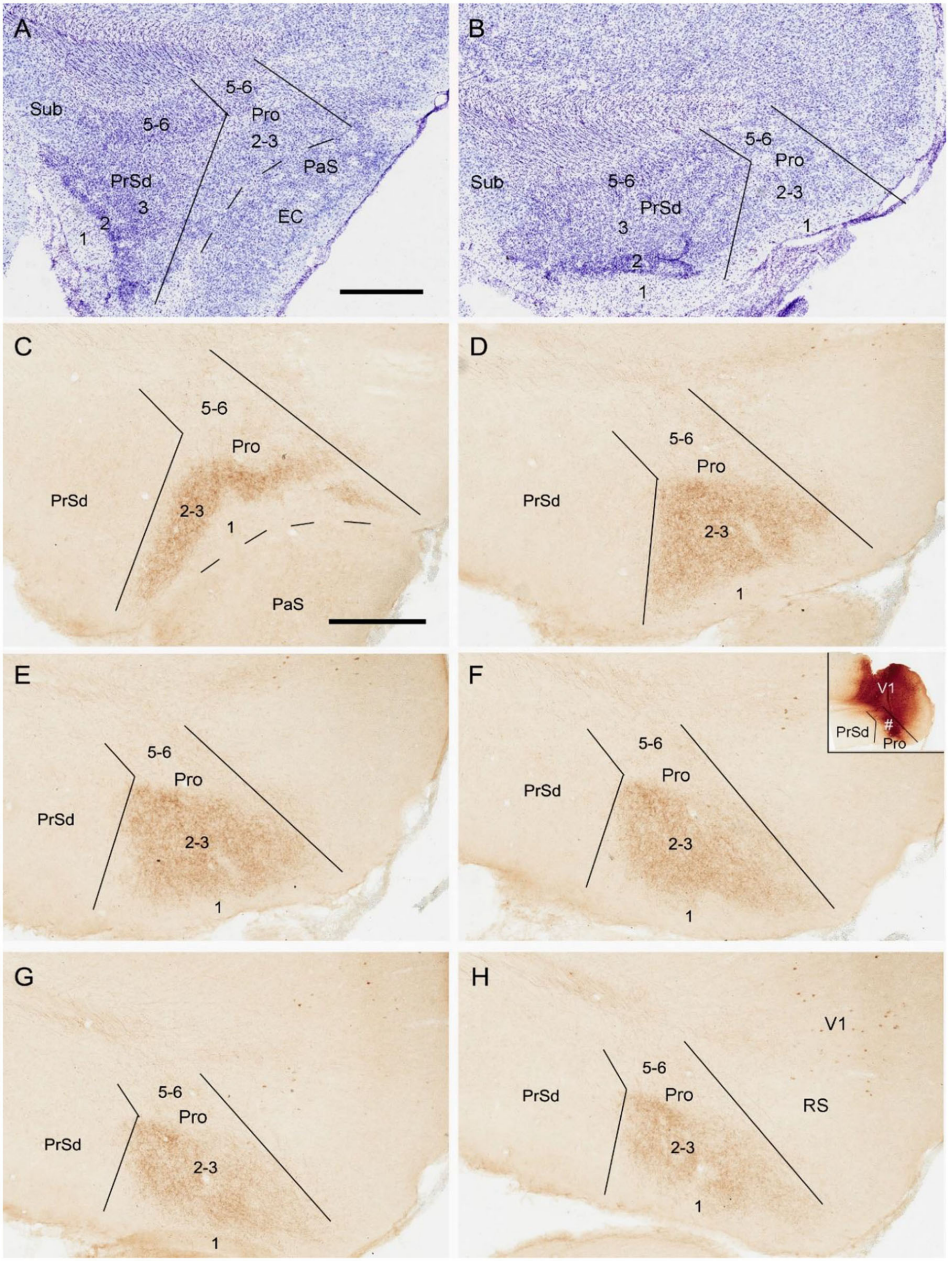
Location and contralateral projections of the prostriata in rat. **(A,B)** Location and lamination of the prostriata in Nissl-stained sections. **(C–H)** A biotinylated dextran amine (BDA) injection in the prostriata resulted in densely labeled axon terminals, mainly in layers 2-3 of contralateral prostriata [showing from lateral **(C)** to medial levels **(H)** in sequential sagittal sections]. The injection site, at about level F of injection side, was involved in both deep and superficial layers of the prostriata (# in the inset of **F**) as well as overlying primary visual cortex (V1) but not in the PrSd. Note that no labeled axon terminals were seen in contralateral PrSd and PaS but sparsely labeled neurons were found in retrosplenial cortex (RS) and V1. Bars: 400 μm in **A** (for **A,B**); 400 μm **C** (for **C–H**).

**Figure 3:**
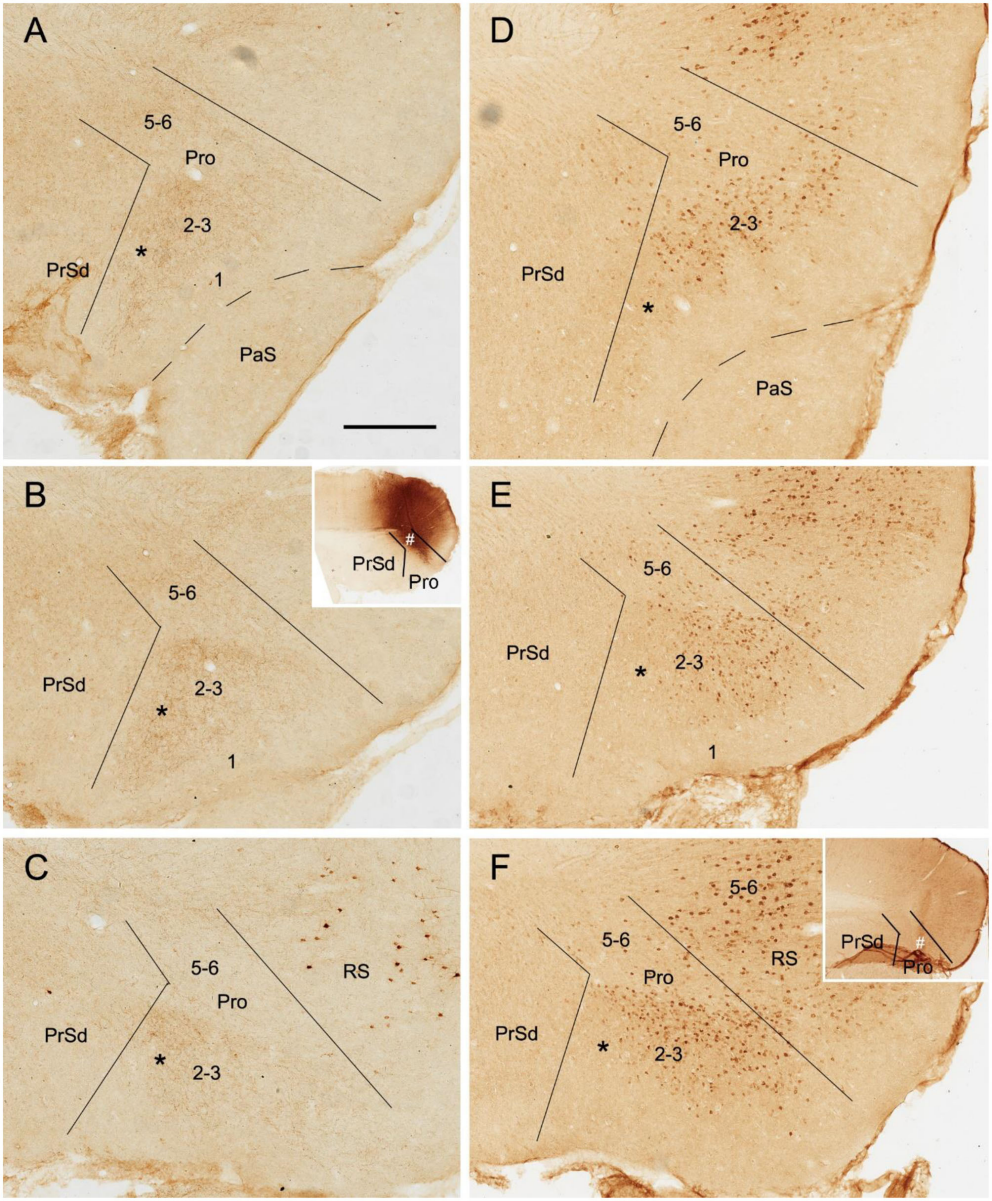
Axon terminals and cells of origin of contralateral projections in rat. **(A–C)** (BDA labeling) and **(D–F)** (FG labeling) are at matched lateral-medial levels. The regions marked by “*” in each row show the corresponding part of the prostriata. **(A–C)** One BDA injection affected layers 5–6 and, slightly, layers 2–3 of the prostriata (# in the inset of **B**) and the overlying V1, resulting in weakly labeled axon terminals in layers 2–3 and 5–6 of contralateral prostriata. The injection, at about level B of injection side, was located slightly more lateral than that shown in Figure 2, and the labeled terminals tend to distribute in the ventral part of contralateral prostriata, marked by “*”. **(D–F)** One Fluoro-Gold (FG) injection affecting both the prostriata (# in the inset of **F**) and the overlying V1 resulted in densely labeled neurons in layers 2–3 of contralateral prostriata. The injection, at about level F of injection side, was located more medial than that shown in Figure 2 and contralaterally labeled neurons tend to distribute in the dorsal part of prostriata rather than the ventral part marked by “*”. Note that many labeled neurons were also seen in the retrosplenial cortex (RS) and V1. Bar: 400 μm in **A** (for all panels).

To reveal the contralateral projection pattern of the prostriata, anterograde tracer BDA was successfully injected into the prostriata of one hemisphere (7 cases). Injection sites were determined based on the location of the rat prostriata demonstrated with Nissl and calbindin stains [Lu et al. (2020); also see Figures 2A,B].The injections were centered in the prostriata with few (3 cases) or some leakage (4 cases) in the overlying primary visual cortex (V1). Each of the injection sites in the prostriata covered almost the entire thickness (5 cases; group 1) or mostly the deep layers (layers 5-6) of the prostriata (2 cases; group 2). Contralaterally, densely labeled axon terminals were only found in the prostriata with few, if any, terminal labeling in MEC, PrSd and PaS in group 1. Labeled axon terminals were restricted to layers 2-3 of the prostriata with no labeling in layers 5-6 (Figures 2C–H). Terminal labeling in layers 2-3 of contralateral prostriata was much sparser in group 2 than in group 1 (Figures 3A–C). Note that no neuronal labeling was observed in the contralateral PrSd and PaS but some scattered neurons were seen in V1 (Figure 2H) and RS (Figure 3C). As a control, BDA injections restricted to V1 were performed. They yielded few, if any, terminal labeling in contralateral prostriata (not shown). These results suggest that the contralateral prostriata projections mostly derive from superficial layers of the prostriata.

### Cells of Origin of Contralateral Projections of the Prostriata in Rats

**Figure 4:**
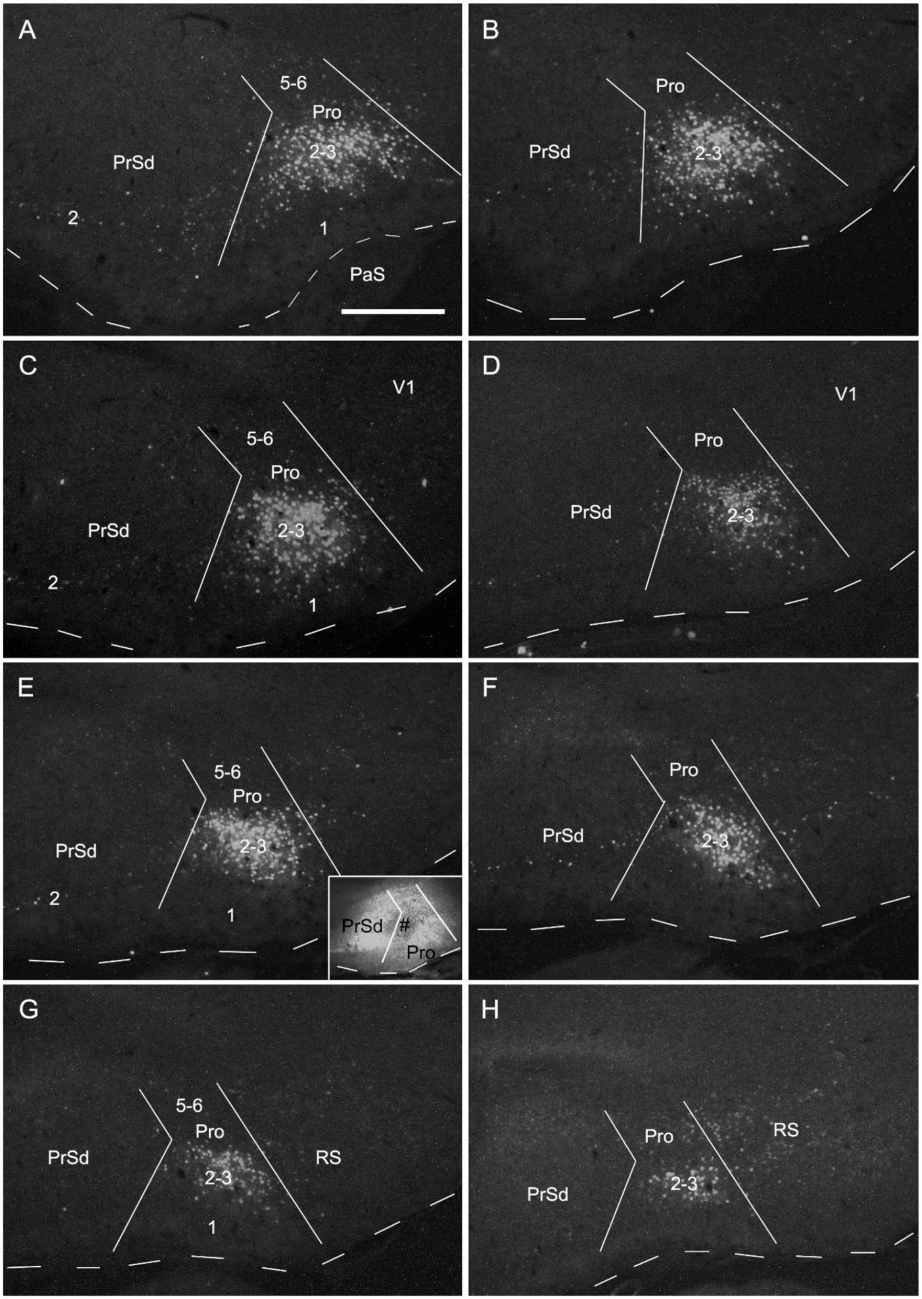
Cells of origin of contralateral projections of the prostriata and PrS in rat. **(A–H)** One FG injection in the prostriata and PrS resulted in neurons being densely labeled in layers 2–3 of prostriata and sparsely labeled in layer 2 of PrS (showing from lateral **(A)** to medial **(H)** levels in sequential sagittal sections). The injection site, at about level E of injection side, was centered in the prostriata (# in the inset in **E**), with some leakage in the PrS and RS but few in V1. Note the labeled neurons in the deep portion of contralateral RS **(H)**. For orientation and shape of each section in this figure please refer to Figure 2. Bar: 500 μm in **A** (for all panels).

To determine the cells of origin in the prostriata, FG injections were successfully placed in one side of the prostriata with some leakage in the overlying V1 (7 cases; group 3) or adjoining PrSd or RS (2 cases; group 4). The injections in group 3 covered almost all layers of the prostriata, and resulted in retrogradely labeled neurons in contralateral prostriata, mostly in layers 2-3 with few in layer 5 (Figures 3D–F). Note that no neurons were labeled in the contralateral PrS and PaS, but some were labeled in RS and V1 (Figure 3). In group 4, when injection sites were situated in prostriata and PrSd (inset in Figure 4E) or RS (not shown), retrogradely labeled neurons in contralateral side were seen densely in layers 2-3 of prostriata and sparsely in layer 2 of PrSd (Figures 4A–H), similar to the results in rat (Preston-Ferrer et al., 2016). Taken together, aforementioned findings suggest the involvement of the injection in ipsilateral PrSd likely results in retrogradely labeled neurons in contralateral PrSd although the possibility of faint PrSd projections to contralateral prostriata cannot be ruled out. Note that some scattered neurons were seen in RS and V1 as well. Furthermore, the contralaterally labeled terminals from the injection located in the lateral (ventral) prostriata tends to distribute in the lateral (ventral) part of the contralateral prostriata (marked with “^*^”, Figures 3A–C). Similarly, contralaterally labeled neurons from the injection located in the medial (dorsal) prostriata tend to distribute in the medial (dorsal) part of the contralateral prostriata (Figures 3D–F). These results suggest possible topographical commissural connections between two sides of the prostriata's layers 2-3.

### Contralateral Projections of the Prostriata in Mice

**Figure 5:**
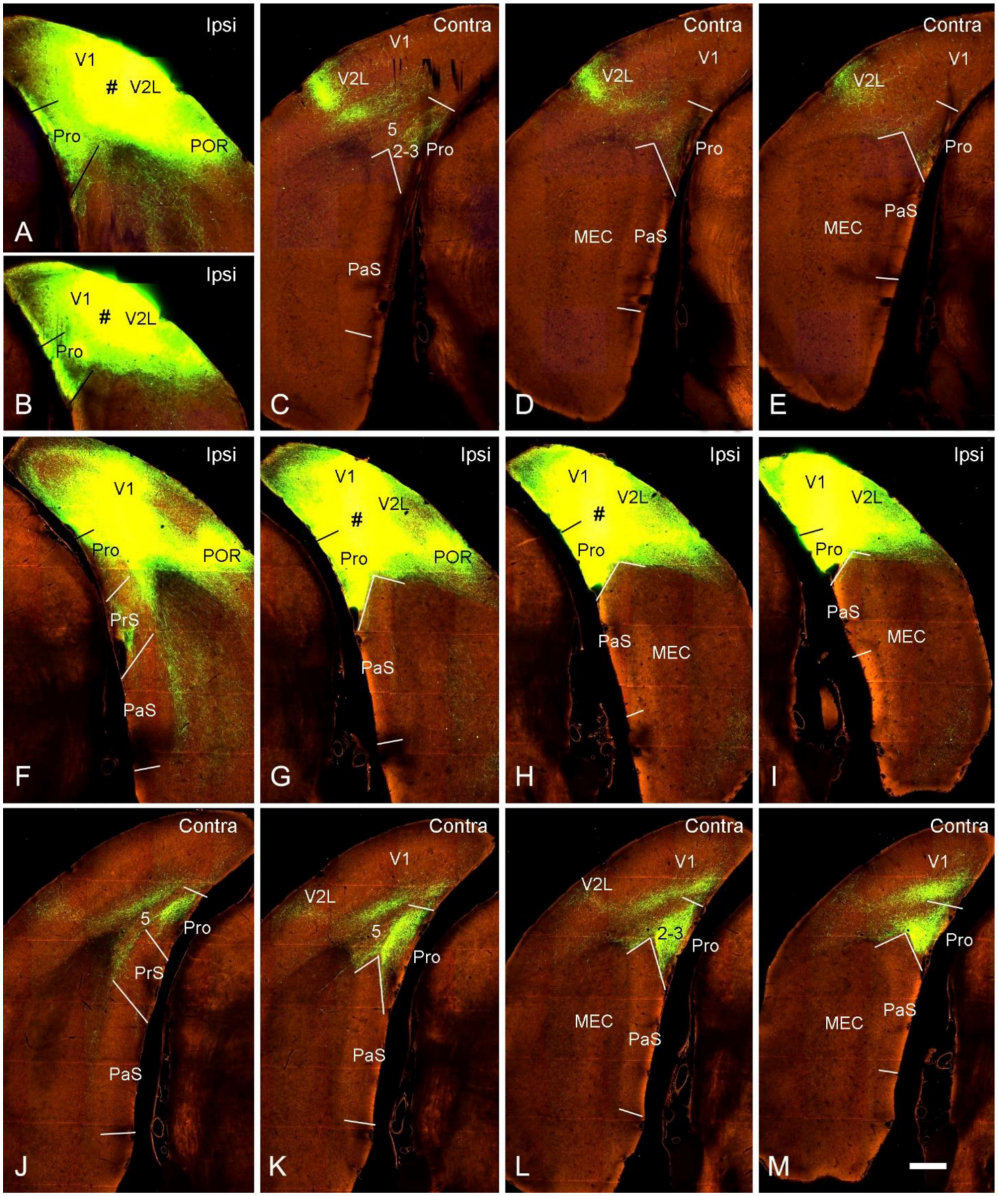
Contralateral projections of the prostriata in wild-type mouse. **(A–E)** An injection located in caudal visual cortex (V1 and V2L; # in **A,B**) resulted in weak and sparse axon terminal labeling in contralateral prostriata (**C–E**; mainly in layers 2-3). No labeling occurred in contralateral MEC, PrS, and PaS, while strong labeling occurred in V2L. **(F–M)** An injection involved in both the caudal visual cortex (V1 and V2L) and the prostriata (# in **G,H**) resulted in strong and dense terminal labeling in the contralateral prostriata **(J–M)**. No labeling was found in the PaS, on either sides, nor in the contralateral MEC. On the ipsilateral side, weak terminal labeling was observed in the MEC (**F–I**; mainly deep layers) and PrSd (**F**; mainly layer 2). Bar: 400 μm in **M** (for all panels).

Contralateral projections of the mouse prostriata were identified by comparing the projection patterns of V1/V2L and V1/V2L+prostriata injections although mouse prostriata is very small and difficult to be targeted. With a large anterograde tracer injection into caudal V1/V2L in one hemisphere of wild-type mouse (Figures 5A,B), weak and sparse terminal labeling was found in layers 2-3 of contralateral prostriata (Figures 5C–E). However, when a comparable injection was applied to both caudal V1/V2L and the full extent of the prostriata (Figures 5F–I), strongly and densely labeled axon terminals were observed in layers 2-3 of full extent of contralateral prostriata (Figures 5J–M). Note that no terminal labeling was seen in superficial layers of contralateral PrS, PaS and MEC in either case. These results suggest that the prostriata itself, rather than V1/V2L, is the source for strong projections to contralateral prostriata.

### Origins of Contralateral Projections of the Prostriata in Mice

**Figure 6:**
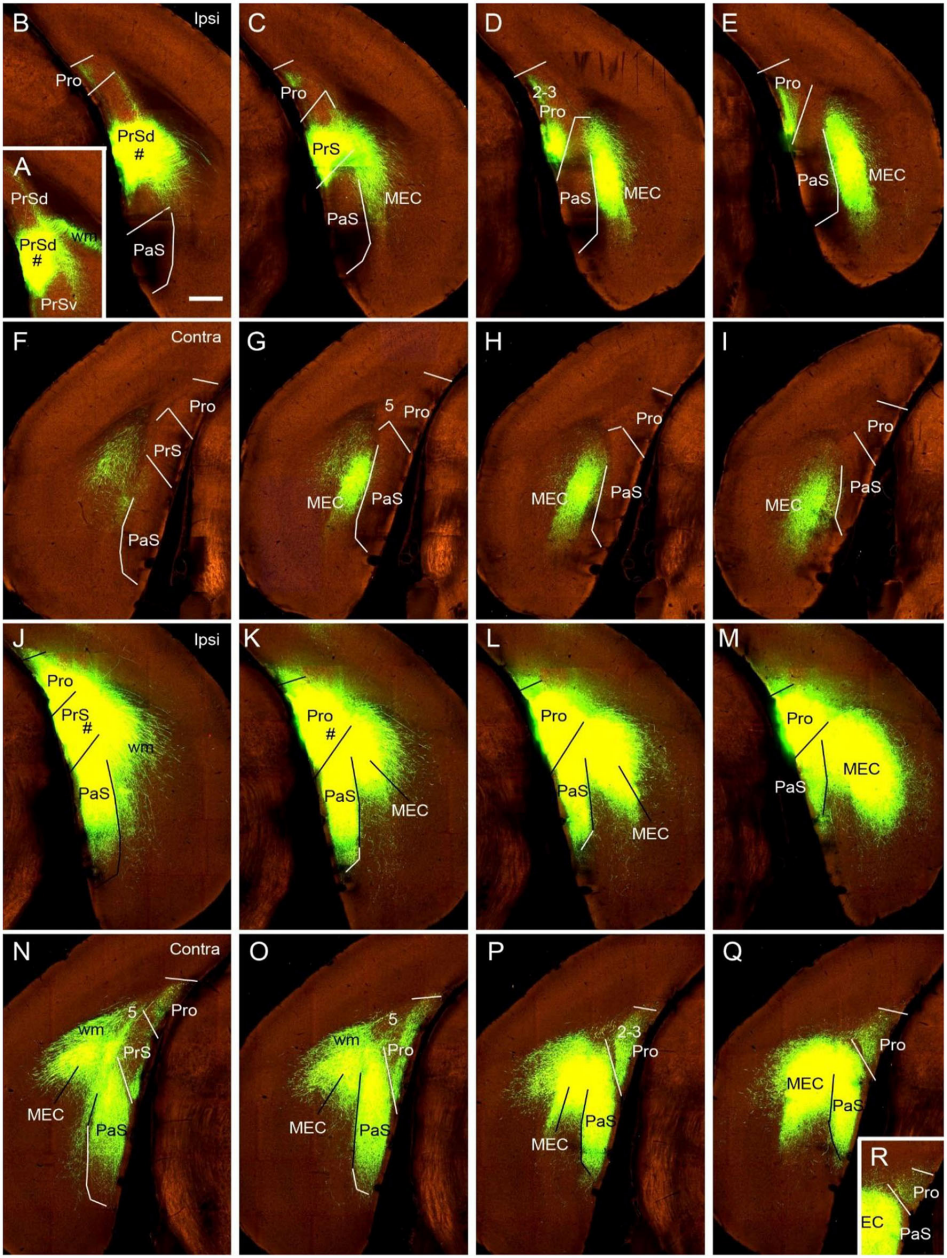
Ipsi- and contralateral projections from prostriata vs. PrS and PaS. Each row shows sequential rostral (left) to caudal (right) coronal sections from *Slc17a6*-IRES-Cre mice. **(A–E)** An injection site in the ventral PrSd (# in **A,B**), and the resulting terminal labeling in the ipsilateral prostriata (mainly in layers 2-3) and the medial entorhinal cortex (MEC; mainly in layers 2-3). Note the lack of labeling in PaS. **(F–I)** Contralateral terminal labeling in MEC, which mirrors the ipsilateral MEC labeling in location and density. No labeling occurred in the contralateral prostriata, PrS, nor the PaS. **(J–M)** An injection site involved in PrSd, PaS and prostriata (# in **J,K**) and the resulting terminal labeling in dorsal MEC **(K–M)**. **(N–R)** Contralateral terminal labeling in prostriata, PaS and MEC. The dense labeling in PaS and MEC mirrors the ipsilateral labeling. Note the strong terminal labeling in contralateral prostriata (**N–R**; mainly in layers 2-3). Bar: 400 μm in **B** (for all panels).

**Figure 7:**
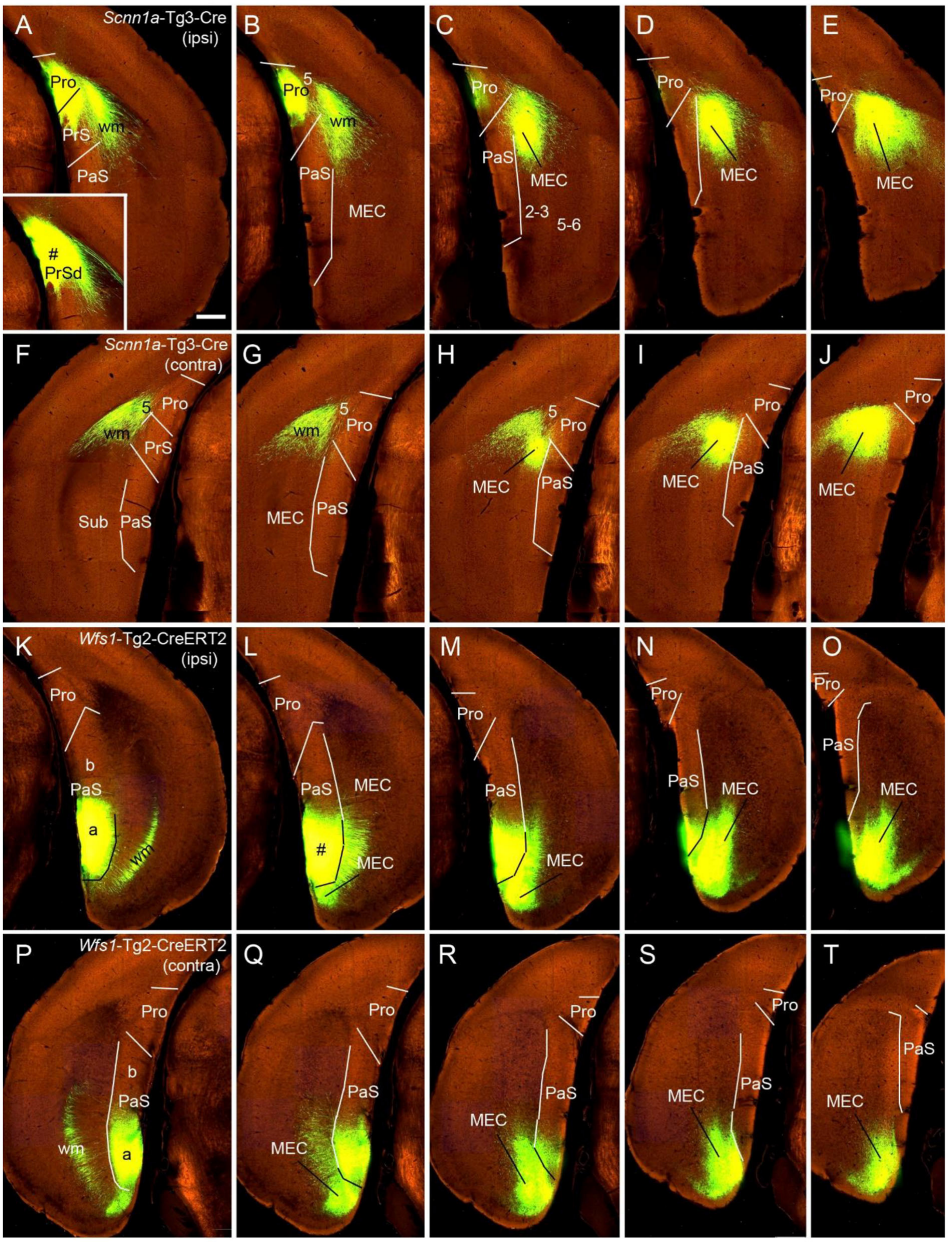
Ipsi- and contralateral projections from PrS and PaS. Each row shows sequential rostral (left) to caudal (right) coronal sections. **(A–E)** An injection site in the dorsal PrSd (# in the inset in **A**) of a *Scnn1a*-Tg3-Cre mouse (the Cre is expressed predominantly in layer 3) and the resulting terminal labeling in the ipsilateral prostriata (mainly in layers 1–3) and the dorsal part of MEC (mainly in layers 2–3) (Figures 6A–E). **(F–J)** Contralateral terminal labeling in MEC, which mirrors the ipsilateral labeling in location and density. Note the lack of labeling in PrS, prostriata and PaS. **(K–O)** An injection site in the ventral PaS (# in **L**) of a *Wfs1*-Tg2-CreERT2 mouse (the Cre is expressed in layers 2–3 of the PaS; see Figures 1P-T) and the resulting terminal labeling in the ipsilateral ventral MEC (mainly in layers 2–3). No labeling was seen in the ipsilateral prostriata and PrS. **(P–T)** Terminal labeling in contralateral PaS and MEC, which mirrors the labeling in the ipsilateral side in location and density. Note the absence labeling in PrS and prostriata. Bar: 400 μm in **A** (for all panels).

To determine if the contralateral projections of the prostriata originate mostly from layers 2-3 of the prostriata, we used *Slc17a6*-IRES-Cre mice. The gene/Cre is expressed in layers 2-3 of the prostriata as well as in the PaS and PrS (see Figures 1K–O), with little expression in layer 2 of the dorsal portion of the PrSd (not shown). For example, when Cre-dependent viral tracers were injected into the ventral portion of the PrSd (Figures 6A,B), no axon terminals were detected in contralateral prostriata (Figures 6F–I) although dense terminal labeling was found in ipsilateral prostriata (Figures 6C–E). In contrast, when an injection was placed in the prostriata, PrS (PrSv) and PaS (Figures 6J–M), strong and dense terminal labeling was observed in layers 2-3 of the contralateral prostriata (Figures 5N–Q). Very strong terminal labeling was also seen in the contralateral MEC and PaS (Figures 6N–Q), probably originating from the PrS/PaS (to MEC) and PaS (to PaS), respectively (see below). Since both PrS and PaS have few, if any, projections to the contralateral prostriata (Figures 6F–I, 7), the strong terminal labeling observed in the contralateral prostriata probably originated from layers 2-3 of the prostriata. This is consistent with the results seen in rat.

### Comparison of Contralateral Projections of Prostriata vs. PrS and PaS in Mice

While the prostriata projects to contralateral prostriata with few projections to contralateral MEC, PrS and PaS, both the PrS and PaS exhibit strong contralateral projections to MEC with few projections to contralateral prostriata (Figures 6A–I, 7). For example, an injection contained in the ventral PrSd (# in Figures 6A,B) or dorsal PrSd (# in the inset of Figure 7A) ipsilaterally resulted in terminal labeling in the prostriata (Figures 6B–E, 7A–E) and the dorsal MEC (Figures 6D,E, 7C–E). In both target regions, labeled terminals from the dorsal PrSd were generally located dorsal to those from the ventral PrSd, suggesting topographical projections. Contralaterally, labeled terminals were seen in the MEC at symmetric locations and no terminals were detected in the prostriata and PrS (Figures 6F–J, 7F–J). For comparison, a single injection placed in the ventral PaS (PaSa; # in Figures 7K,L) of a *Wfs1*-Tg2-CreERT2 mouse (Cre is strongly expressed in layers 2-3 of the PaS; see Figures 1P–T) or dorsal PaS (PaSb; not shown) ipsilaterally produced strong terminal labeling in both MEC and PaS with no labeling in the prostriata (Figures 7M–O). The same is true for contralateral projections at symmetric locations (Figures 7P–T). In both MEC and PaS, bilaterally labeled terminals from PaSb injections were generally located dorsal to those from PaSa injections, suggesting topographical projections of the PaS. As for the laminar distribution of labeled terminals, PrS and PaS projections largely terminate in layers 3 and 2 of the MEC, respectively. Commissural projections from the PrS (PrSd and PrSv) mainly target layer 2 of contralateral PrS with weak labeling in layer 5 while those from the PaS target layers 2-3 of the PaS.

To determine the cells of origin of the commissural connections of the PrS, we compared two Cre-dependent tracing experiments with injections in PrS. When an injection was placed in the PrS of a *Drd3*-Cre_KI196 mouse, in which the *Drd3*-Cre was expressed in layer 3 but not in layer 2 of the PrS, no terminal labeling was observed in contralateral PrS (not shown). In contrast, an injection in the PrS of a *Grm2*-Cre_MR90 mouse resulted in terminal labeling in layers 2 (moderate) and 5 (weak) of the contralateral PrS. In this mouse the *Grm2*-Cre was expressed in layers 2 and 3 of the PrS (not shown). It should also be noted that injections placed in the PrSd of a *Slc17a6*-IRES-Cre mouse (Figures 6A,B) and a *Scnn1a*-Tg3-Cre mouse (inset in Figure 7A) did not produce clearly labeled terminals in contralateral PrS. These findings indicate that layer 2 of the PrS is the main origin of the commissural connections of the PrS.

## Discussion

**Figure 8:**
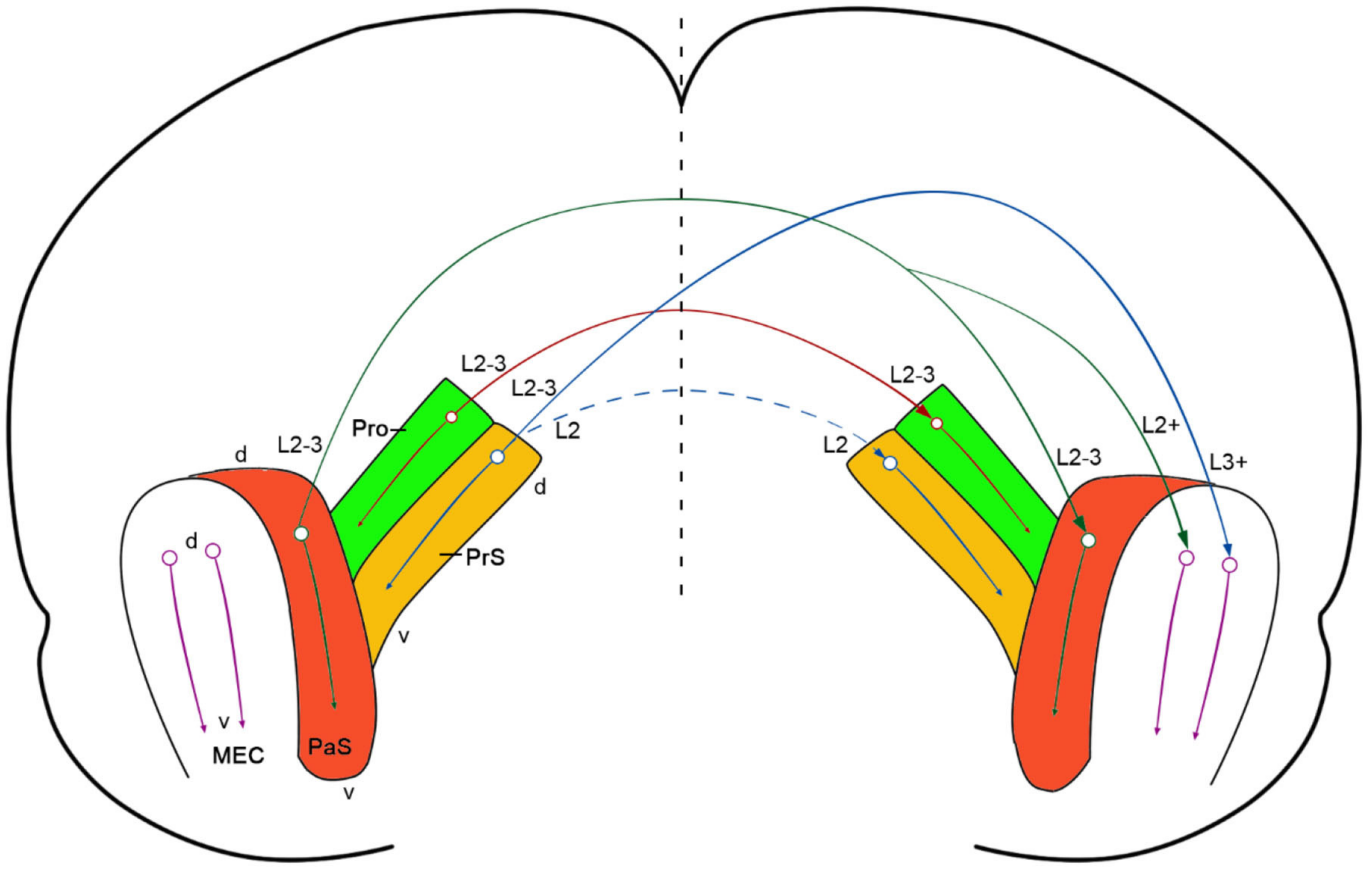
Summary and comparison of commissural connections of the prostriata, PrS and PaS. A diagram showing strong prostriata projections to contralateral prostriata (solid red arrow) and strong PrS and PaS projections to contralateral MEC (solid blue and solid green arrows, respectively). The strong and weak homotopic commissural projections of the PaS (solid green arrow) and PrS (dashed blue arrow) are also indicated, respectively. The prostriata, PrS, and PaS in both hemispheres are color coded green, brown, and red, respectively (MEC was not color coded). The circle-arrows in each structure indicate dorsal-ventral orientation and topographical projections. The major layers of the cells of origin and axon terminations are also marked. The vertical dashed line indicates midline.

In this study, we have revealed the existence of strong commissural connections between bilateral prostriata of the rat with both anterograde and retrograde tracing methods and confirmed this finding in mouse with Cre-dependent tracing (Figure 8). These commissural connections almost always originate from layers 2-3 of the prostriata and project to layers 2-3 of contralateral prostriata with few (if any) to other regions beyond the prostriata, making them almost “pure” homotopic interhemispheric projections (with no or few heterotopic projections). In literature, interhemispheric projections are mainly mediated by callosal projections in mammalian brains and these projections are both homotopic and heterotopic (Záborszky and Wolff, 1982; Miller and Vogt, 1984; Ding and Elberger, 2001; Huang et al., 2013; Restani and Caleo, 2016). Callosal projections were reported to play important roles in interhemispheric integration, coordination and the balancing of information (van der Knaap and van der Ham, 2011; Shen et al., 2015; Zhang et al., 2015; Restani and Caleo, 2016). In rat and mouse V1, callosal projecting neurons are mostly located at the V1/V2L border and in the lateral portion of the V1 (Miller and Vogt, 1984; Olavarria and Montero, 1989; Ding and Elberger, 2001), which represents the central visual field. In contrast, neurons in the medial portion of V1, which represents the peripheral visual field, send much fewer callosal projections (Záborszky and Wolff, 1982; Miller and Vogt, 1984; Restani and Caleo, 2016). Recently the medial portion of V1 has been found to send direct and strong projections to the prostriata (Lu et al., 2020). Our main finding in this study that layers 2-3 of the prostriata have strong homotopic commissural connections suggest the prostriata is a unique region for fast processing and integration of bilateral information from peripheral visual space. Therefore, information from peripheral visual fields could reach the prostriata via direct projections from medial V1 to the prostriata, where interhemispheric integration and coordination of related information could occur. The prostriata has been suggested to play a role in monitoring peripheral visual field for new, unexpected, and especially moving, stimuli (Yu et al., 2012; Mikellidou et al., 2017; Tamietto and Leopold, 2018). It is hypothesized that dangerous moving object(s) appearing in the peripheral visual space of both sides could be quickly processed and integrated in the prostriata via its homotopic interhemispheric connections, enabling proper and coordinated adaptive behaviors.

Previous studies in rat revealed strong commissural connections of the PrS and PaS (van Groen and Wyss, 1990; Honda et al., 2008). Specifically, layer 2 of the PrS has projections to the contralateral layers 2 and 5 of the PrS (Honda et al., 2008). Similarly, layer 2-3 of the PaS was reported to have commissural projections to layers 2-3 of the contralateral PaS (van Groen and Wyss, 1990). The present study has revealed similar findings in mouse using Cre-dependent viral tracers. When these tracers were restricted to layers 2-3 of the PaS of the *Wfs1*-Cre mice, in which the Cre is only expressed in layers 2-3 of the PaS (Figures 1P–T), labeled terminals in the contralateral side were present in layers 2-3 of the PaS as well as in layer 2 of the MEC while no labeling occurred in the prostriata and PrS (e.g., Figures 7P–T).

Homotopic commissural connections are found between both sides of the prostriata, PrS, and PaS. However, distinct differences exist with regard to heterotopic commissural connections (Figure 8). In contrast to the prostriata, which does not show clearly detectable projections to contralateral MEC, both PrS and PaS have strong projections to contralateral as well as ipsilateral MEC in mouse (this study) and rat (van Groen and Wyss, 1990; Caballero-Bleda and Witter, 1993; Honda et al., 2008). Therefore, unlike the PrS and PaS, the prostriata may not have significant impact on the activities of the neurons which process topographic spatial information, such as grid and head-direction cells in layers 2-3 of the MEC (Gu et al., 2018; Rowland et al., 2018). The differential connections of the prostriata vs. the PrS and PaS also support the proposition that the prostriata in rodent is a distinct anatomical entity from adjoining regions (Ding, 2013; Lu et al., 2020). This is also consistent with their differential functions. For example, the prostriata was reported to be critical to fast detection and analysis of peripheral moving visual stimuli (Yu et al., 2012; Mikellidou et al., 2017; Tamietto and Leopold, 2018) while the PrS and PaS were found to be important for episodic memory and spatial navigation (Boccara et al., 2010; Preston-Ferrer et al., 2016; Tang et al., 2016; Dalton and Maguire, 2017).

## Data Availability Statement

The original contributions presented in the study are included in the article/supplementary materials, further inquiries can be directed to the corresponding author/s.

## Ethics Statement

The animal study was reviewed and approved by IACUC of Guangzhou Medical University.

## Author Contributions

S-LD: conceptualization. C-HC, J-MH, and S-LD: investigation and analysis. C-HC and S-LD: writing. S-LD, S-QC, and S-ML: supervision. All authors contributed to the article and approved the submitted version.

## Conflict of Interest

The authors declare that the research was conducted in the absence of any commercial or financial relationships that could be construed as a potential conflict of interest.
